# Screening Heroin Smokers Attending Community Drug Clinics for Change in Lung Function: A Cohort Study

**DOI:** 10.1016/j.chest.2019.11.006

**Published:** 2019-11-22

**Authors:** Rebecca Nightingale, Kevin Mortimer, Emanuele Giorgi, Paul P. Walker, Marie Stolbrink, Tara Byrne, Kerry Marwood, Sally Morrison-Griffiths, Susan Renwick, Jamie Rylance, Hassan Burhan

**Affiliations:** a Liverpool School of Tropical Medicine, Liverpool, England; b CHICAS, Lancaster University, England; c University Hospital Aintree, Liverpool, England; d Addaction, Liverpool, England; e Liverpool Clinical Commissioning Group, Liverpool, England; f Malawi-Liverpool-Wellcome Trust Clinical Research Programme, Blantyre, Malawi; g Royal Liverpool and Broadgreen University Hospitals, Liverpool, England

**Keywords:** COPD, heroin, opiate, spirometry, ACO, asthma-COPD overlap, ATS, American Thoracic Society, CAT, COPD Assessment Tool, GOLD, Global Initiative for Chronic Obstructive Lung Disease, MRC, Medical Research Council Dyspnea Scale, OST, opiate substitution therapy

## Abstract

**Background:**

Heroin smokers have high rates of COPD, respiratory morbidity, hospital admission, and mortality. We assessed the natural history of symptoms and lung function in this population over time.

**Methods:**

A cohort of heroin smokers with COPD was followed for 18 to 24 months. At baseline and follow-up, respiratory symptoms were measured by the Medical Research Council Dyspnea Scale (MRC) and the COPD Assessment Tool (CAT), and postbronchodilator spirometry was performed. Frequency of health-care-seeking episodes was extracted from routine health records. Parametric, nonparametric, and linear regression models were used to analyze the change in symptoms and lung function over time.

**Results:**

Of 372 participants originally recruited, 161 were assessed at follow-up (mean age, 51.0 ± 5.3 years; 74 women [46%]) and 106 participants completed postbronchodilator spirometry. All participants were current or previous heroin smokers, and 122 (75.8%) had smoked crack. Symptoms increased over time (MRC score increased by 0.48 points per year, *P* < .001; CAT score increased by 1.60 points per year, *P* < .001). FEV_1_ declined annually by 90 ± 190 mL (*P* < .001). This deterioration was not associated with change in tobacco or heroin smoking status or use of inhaled medications.

**Conclusions:**

Heroin smokers experience a high and increasing burden of chronic respiratory symptoms and a decline in FEV_1_ that exceeds the normal age-related decline observed among tobacco smokers with COPD and healthy nonsmokers. Targeted COPD diagnostic and treatment services hosted within opiate substitution services could benefit this vulnerable, relatively inaccessible, and underserved group of people.

FOR EDITORIAL COMMENT, SEE PAGE 484

Illicit drug use is common, with 8.5% of adults in England and Wales having reported taking an illicit drug in 2016 and 2017.^1^ Over the last 30 years, smoking rather than injecting heroin has become more common.^2^, ^3^, ^4^, ^5^, ^6^ In recent years, smoking heroin rather than injecting has been used as a possible method of harm reduction.^7^^,^^8^

Although the effects of illicit drug use are well documented, there is limited evidence about the chronic effects of inhaled illicit drug use on the respiratory system. Multiple case reports highlight acute asthma attacks in heroin users, and observational studies report a high prevalence of respiratory disease in heroin users admitted to acute hospitals.^9^^,^^10^ Severe early onset emphysema associated with premature mortality has been reported among heroin users.^11^, ^12^, ^13^ However, large-scale diagnostic studies in this hard-to-reach population are lacking. Chronic respiratory symptoms are common in people inhaling heroin; however, access to formal diagnosis including lung function measurement is limited.^14^, ^15^, ^16^

We recently reported postbronchodilator spirometry in 703 heroin smokers attending for opiate substitution therapy (OST) at community drug service clinics in Liverpool, England; 50% of heroin smokers had either COPD or asthma-COPD overlap (ACO) despite a mean age of 47 years.^17^ This was associated with extensive respiratory symptoms, which given the known high rates of COPD hospitalization and a continuing trend toward inhalation as the mode of drug use, is likely to put increased burden on health systems.^4^^,^^6^^,^^18^ In light of this, screening and treatment programs for heroin smokers could be a viable method for identifying and treating disease in this relatively inaccessible patient group.^19^

We performed a longitudinal cohort study of heroin smokers attending community drug services and who were recruited as an original cohort of 703 heroin smokers described in terms of baseline characteristics in our previous paper.^17^ The aim was to ascertain their change in health status, respiratory symptoms, and lung function over an 18- to 24-month period.

## Methods

### Setting

The study was performed in 31 community drug service clinics in Liverpool. Clinics are run by Addaction, a large independent charity commissioned by the local city council public health department. A keyworker who knew the client and who coordinated their OST worked with the study team in each clinic.

### Participants

Participants were invited to take part if they had previously completed spirometry in the baseline screening project that took place between December 2015 and June 2016,^17^ were > 18 years of age, and were still fully enrolled in Addaction’s service. All participants were current or previous smokers of heroin and were currently or recently treated with methadone or buprenorphine. Participants were given the study information prior to being booked for their regular appointment and were offered a study visit at their usual clinic. People missing their usual appointment were offered another at a central venue. Written informed consent was obtained from all participants.

### Variables and Data Source

Baseline data collection has been previously described.^17^ In brief, participants completed a questionnaire detailing demographic data, and self-reported tobacco and illicit drug use. Oxygen saturations were measured, and pre- and postbronchodilator spirometry was completed.

At follow-up, participants completed a questionnaire which evaluated self-reporting medication prescriptions, health-care access, and ongoing tobacco and illicit drug use. The index of multiple deprivation, which is an official geographic measure of relative deprivation in England, was used a proxy of social-economic status.^20^ Participants also completed the COPD Assessment Tool (CAT)^21^ and the Medical Research Council Dyspnea Scale (MRC),^22^ and consented to allow review of 2 years of medical records for respiratory-related diagnosis and prescriptions from primary care pharmacy records (EMIS), and hospital records where applicable.

Oxygen saturations were measured, and pre- and postbronchodilator spirometry was performed on all participants who consented and did not have medical contraindications. Spirometry was performed by trained clinical staff and completed according to American Thoracic Society (ATS) guidelines.^23^ All traces were double-reviewed for quality and grading by an experienced respiratory physician. As with the baseline survey, participants were asked not to take a short-acting bronchodilator within 8 h of visit or a long-acting bronchodilator within 24 h. If they had taken a short-acting inhaler, only postbronchodilator spirometry was recorded.

Subjects were categorized based on original screening. A diagnosis of asthma was given if airflow obstruction (FEV_1_/FVC ratio < 0.7) was fully reversible to inhaled salbutamol (ie, either FEV_1_/FVC normalized or FEV_1_ increased by ≥ 400 mL), or if spirometry was normal but the participant had a prior physician diagnosis of asthma. Participants with nonreversible airflow obstruction were characterized as COPD unless they had a prior physician diagnosis of asthma, in which case their condition was labeled as ACO. We report the lung function change of participants who had been diagnosed with COPD or ACO at baseline; participants with an asthma diagnosis were excluded.^17^

All spirometry data were reported using the European Coal and Steel Community^24^ reference ranges for consistency with prior work. Abnormal spirometry was defined using the Global Initiative for Chronic Obstructive Lung Disease (GOLD).^25^ Change in lung function was based on postbronchodilator FEV_1_.

### Sample Size

We aimed to follow-up as many of the participants with COPD or ACO from baseline as possible.

### Statistical Analysis

Univariate analysis was carried out using descriptive statistics to explore the characteristics of the study populations. Paired *t* tests and Wilcoxon sign rank tests (with bootstrapping to estimate the CI of the difference) were used to assess change between the two time points. Time was used as a continuous variable to account for variation between follow-up dates and to calculate an annualized change. A linear regression model was used to estimate the effect of potential factors (change in inhaled illicit drug use, change in tobacco smoking, and change in inhaler use) on changes in FEV_1_ over time. Variables were selected for the model a priori based on clinical data which might have varied over the course of follow-up within an individual, specifically in participants who described changes in drug or medication use. The whole model is presented without variable elimination. Data were analyzed using Stata version 14.2 statistical software (StataCorp LLC) and R version 3.4 (R Foundation for Statistical Computing). Statistical significance was tested at the conventional 5% level.

### Ethics

Ethical approval was gained from the Health Research Authority via the Integrated Research Application System (No. 235151).

## Results

**Figure 1:**
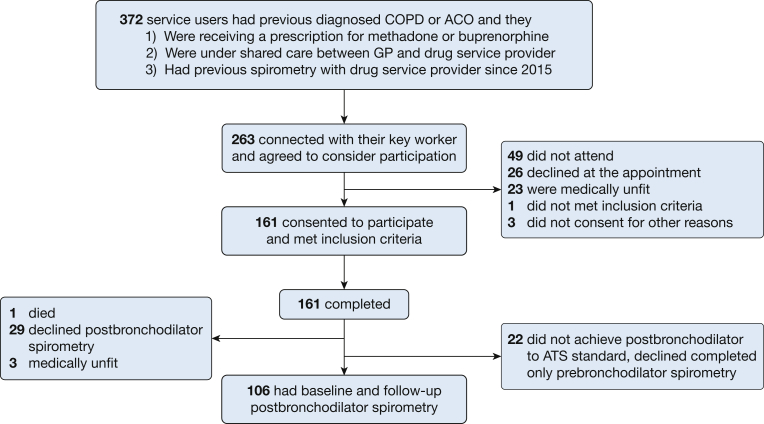
Flow of participants through the study. ACO = asthma-COPD overlap; ATS = American Thoracic Society; GP = general practitioner.

A total of 372 participants had previous COPD or ACO and were eligible for inclusion. The study follow-up took place between December 2017 and April 2018. Baseline questionnaire and clinical data were collected from 161 participants; 109 were lost to follow-up, 49 did not attend the follow-up appointment, 26 declined at the appointment, 23 were medically unfit, and four did not take part for other reasons. A total of 106 participants completed postbronchodilator spirometry at both baseline and follow-up to ATS standards. Patients remaining (n = 55) did not meet ATS standards (n = 22), were medically unfit (n = 3), died (n = 1), or declined postbronchodilator spirometry (n = 29) (Fig 1). Compression of participants characteristics can be seen in e-Table 1.

The characteristics of the population are given in Table 1. Participants had a mean age of 51 ± 5.3 years, and 46 (28.6%) were women. Most participants were unemployed with high levels of socioeconomic deprivation (mean index of multiple deprivation score 51.5, which is in the lowest quintile for England). All participants were taking OST, with 76 (47.2%) reporting current heroin use.

**Table 1:** Characteristics of 161 People With Baseline COPD or Asthma-COPD Overlap Derived From Follow-Up Questionnaire Data

Characteristic	Value
Sex, female	46 (28.6)
Age, y	51.0 ± 5.3
IMD score	51.5 ± 12.7
Occupation	
Unemployed	137 (85.1)
Employment	24 (14.9)
Housing	
Own home (including rented)	124 (77.0)
Homeless	6 (3.7)
Other	31 (19.3)
Cigarette smoking status	
Current	133 (82.6)
Ex	27 (16.8)
Never	1 (0.6)
Cigarettes smoked per day	11 ± 7.0
Heroin smoking status	
Current	76 (47.2)
Ex	85 (52.8)
Bags smoked per week^a^	4.0 ± 7.0
Crack smoking	
Current	33 (20.5)
Ex	89 (55.3)
Never	39 (24.2)
Rocks smoked per week	2.18 ± 1.4
Cannabis smoking status	
Current	38 (23.8)
Ex	53 (33.1)
Never	69 (43.1)
Cannabis joint per week	12 ± 17.1
Ever injected heroin	30 (18.5)
Current methadone dosage, mL/d	45.7 ± 21.6
Current buprenorphine dosage, mg/d	10.4 ± 8.8

Values are presented as mean ± SD or No. (%). IMD = index of multiple deprivation.

a A bag is estimated to equate to 0.1 g.

**Figure 2:**
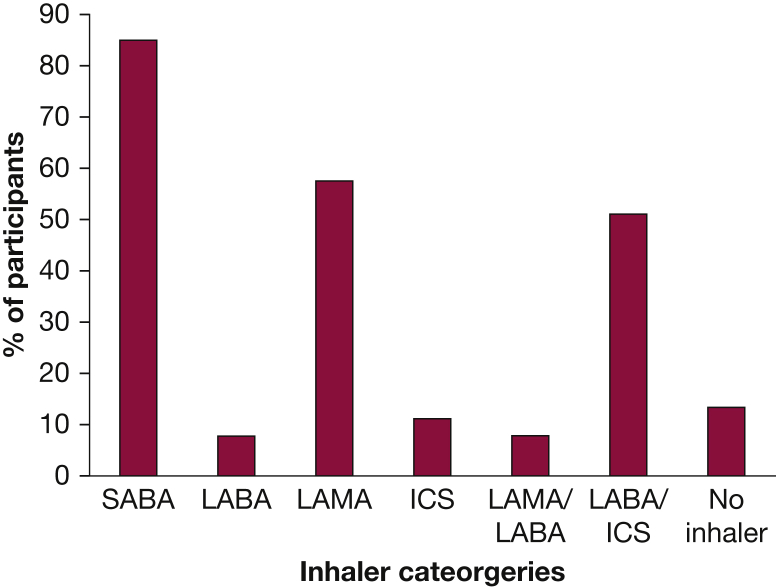
Participants prescribed and picking up their inhalers (at least 50% of what was expected as recorded by the pharmacy team) as recorded on the primary care electronic prescribing system. Inhalers reviewed were SABA, LABA, LAMA, and ICS. ICS = inhaled corticosteroid; LABA = long-acting beta 2 agonist; LAMA = long-acting muscarinic antagonist; SABA = short-acting beta 2 agonist.

Most were prescribed an inhaler and were collecting prescriptions (defined as at least 50% pickup rate) from a pharmacy (n = 131; 81.4%). No inhalers were prescribed or collected for 21 participants (13.3%), and data were unavailable for 9 participants (5.5%). Of the participants with available data, 129 (84.9%), 88 (57.9%), and 78 (51.3%) collected prescriptions for short-acting beta 2 agonist, long-acting muscarinic antagonist, and an inhaled corticosteroid/long-acting beta 2 agonist combination, respectively (Fig 2). Three-quarters had attended a primary care practitioner for respiratory complaints within the preceding 2 years, with 18 (11%) requiring admission to hospital, staying for a mean of 11.5 days. Participants admitted to hospital were universally treated with bronchodilators, antibiotics, and steroids; three participants were offered noninvasive ventilation; two were treated in high-dependency areas; and none had level 3 care (invasive ventilation) (Table 2).

**Table 2:** Health-Care Utilization From 2 Years Prior to Follow-Up Among Those Who Completed Follow-Up Questionnaires (N = 161)

Variable	Value
Taking an inhaler regularly	
Yes	131 (81.4)
No	21 (13.0)
Not known	9 (5.6)
Reported GP visits in last 2 y for respiratory conditions	
Yes	121 (75.2)
No	25 (15.5)
Not known	15 (9.3)
No. of primary care visits (GP or nurse)	8.6 ± 7.0
Emergency hospital visits for respiratory conditions	
Yes	17 (10.6)
No	114 (70.8)
Not known	30 (18.6)
Emergency hospital visits of those who did attend	2.6 ± 1.9
Admitted to hospital in last 2 y for respiratory conditions	
Yes	17 (10.5)
No	121 (74.7)
Not known	24 (14.8)
Length of hospital stay, d	11.5 ± 13.0

Values are presented as No. (%) or mean ± SD. Data were gathered from electronic medical records; participants not appearing on these systems are coded as not known, but might engage with extraregional, informal, or private health-care providers. GP = general practitioner.

The mean FEV_1_ was 2.05 ± 0.96 L at follow-up compared with 2.23 ± 0.97 L at baseline. Of the participants diagnosed with COPD/ACO at baseline and postbronchodilator spirometry at both time points, 94 (88.7%) had spirometry indicative of COPD at follow-up, with 38 (35.9%) having severe or very severe COPD (using GOLD guidelines) at follow-up compared with 26 (24.6%) at baseline. A further five participants (4.7%) had full reversibility (> 400 mL) and therefore were diagnosed with asthma, and seven (6.6%) had normal spirometry at follow-up (Table 3).

**Table 3:** Diagnosis and Postbronchodilator Spirometry at Baseline and 2-Year Follow-Up of the 106 Participants Diagnosed With COPD or ACO at Baseline Who Completed Follow-Up

Variable	Baseline	Follow-Up
FEV_1_, L	2.23 ± 0.97	2.05 ± 0.95
FEV_1_, % predicted	69.1 ± 2.6	64.6 ± 2.7
FVC, L	4.07 ± 1.2	3.69 ± 1.1
FVC, % predicted	102.7 ± 23.7	95.5 ± 23.4
FEV_1_/FVC ratio	0.54 ± 0.13	0.53 ± 0.14
Diagnosis (GOLD)		
ACO	4 (3.8)	…
Asthma	…	5 (4.7)
Normal	…	7 (6.6)
Severity (GOLD)		
Mild	37 (34.9)	23 (21.7)
Moderate	39 (36.8)	33 (31.1)
Severe	15 (14.2)	24 (22.7)
Very severe	11 (10.4)	14 (13.2)

Values are presented as No. (%) or mean ± SD. ACO = asthma-COPD overlap; GOLD = Global Initiative for Chronic Obstructive Lung Disease.

**Figure 3:**
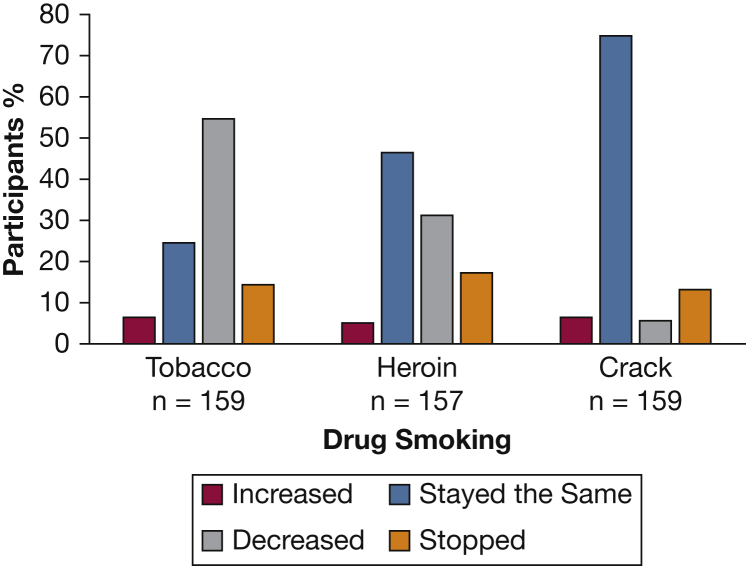
Change in daily consumption of tobacco, heroin, and crack in 161 subjects over 2 y. If they have never smoked, their smoking status was recorded as stayed the same.

Participants reported a significant annualized increase in respiratory symptoms with the MRC and CAT scores increasing by a median of 0.48 (*P* < .001) and 1.60 (*P* < .001), respectively. They experienced a significant annualized decline in FEV_1_ and median oxygen saturation of 90 mL (*P* < .001) and 0.92% (*P* < .001), respectively (Table 4). Changes in smoking status and inhaler use were prehypothesized possible clinical factors that could influence FEV_1_ change. Since baseline, 49 participants (31.2%) reported a decrease in heroin smoking, and 73 (46.5%) reported unchanged usage (Fig 3). Change in drug use was not associated with change in FEV_1_. The final model showing change in drug and tobacco smoking status and inhaler use is presented in Table 5.

**Table 4:** Annualized Change in Spirometry and Symptoms in the 106 Participants Diagnosed With COPD or ACO at Baseline Who Completed Follow-Up

Variable	Baseline	Follow-Up	Change Per Year	Bootstrapping/95% CI	*P* Value
FEV_1_, L	2.23 ± 97.12	2.05 ± 95.60	−0.09 ± 0.19	−0.05 to −0.13	< .001
MRC score	3 (2-4)	4 (3-5)	0.46 (0.0 to 1.0)	0.52 (0.36 to 0.67)	< .001
CAT score	25 (17-31)	29 (23-33)	1.60 (−0.48 to 4.32)	0.46 (0.29 to 0.60)	< .001
Spo_2_ (%)	97 (96-98)	95 (93-96)	−0.92 (−1.63 to 0.0)	0.53 (0.38 to 0.66)	< .001

Values are mean ± SD, median (25th percentile-75th percentile), or as otherwise indicated. CAT = COPD Assessment Tool; MRC = Medical Research Council Dyspnea Scale; Spo_2_ = peripheral capillary oxygen saturation. See Table 3 legend for expansion of other abbreviation.

**Table 5:** Linear Regression Model of Postbronchodilator FEV_1_ Change (n = 106)

Variable	Coefficient (95% CI) for FEV_1_ Decrease (mL/y)	*P* Value (95% CI)
Change in reported heroin consumption		
No change	Ref	
Increase^a^	5.92	0.36 (−3.46 to 15.31)
Decrease^b^	5.35	0.21 (−6.31 to 17.03)
Change in reported crack consumption		
No change	Ref	
Increase^a^	0.18	0.96 (−9.00 to 7.68)
Decrease^b^	2.69	0.69 (−10.55 to 15.94)
Change in tobacco consumption		
No change	Ref	
Increase^a^	7.81	0.80 (−9.91 to 7.68)
Decrease^b^	−1.11	0.34 (−8.51 to 24.14)
Change in inhaler use		
No change		
Increase^a^	−3.20	0.48 (−12.12 to 5.72)
Decrease^b^	1.79	0.79 (−11.61 to 15.20)

Ref = reference.

a A positive change is an increase in use since baseline.

b A negative change is a decrease in usage since baseline.

## Discussion

In a population of heroin smokers, we found a high burden of lung disease. In the previously published baseline data, 50% of heroin users had COPD or ACO, with a mean MRC score of 3.1 and CAT score of 22.9.^17^ At follow-up, participants’ respiratory symptoms had worsened significantly from baseline, with annual increases in both CAT score (1.60) and MRC score (0.46), and mean oxygen saturation dropping from 97% to 95% from baseline to follow-up. We found that lung function measured by FEV_1_ declined by 90 mL annually, which was both statistically and clinically significant. The proportion of subjects classified as having severe or very severe disease increased from 25% to 36% over the 2-year follow-up period. Neither ongoing illicit drug use nor prescriptions of inhaled medication were associated with change in lung function.

The symptoms reported in this study are consistent with those of studies in this population, with increased dyspnea among heroin users being the common symptom.^12^^,^^13^^,^^26^ The decline in health status measured by a CAT score increase of 1.60 annually is greater than the 1 unit change seen in patients with stable COPD.^27^ The rate of decline in FEV_1_ is considerably higher than both the 30 mL/y age-related decline seen in nonsmokers and in people with tobacco-related COPD (which is reported at 35-79 mL/y, of which all but one paper reported an annual decline of ≤ 69 mL).^26^^,^^28^ To date, research on lung function in heroin smokers has focused on cross-sectional studies. The results from this longitudinal cohort study support and enhance previous cross-sectional studies that suggest heroin users are at a high risk of COPD and suggest that their decline is worse than that of tobacco smokers. Walker et al^11^ found heroin smokers developed early onset emphysema, with a mean age of diagnosis being 41 years, suggesting likely early progression of disease compared with nonheroin smokers. In Amsterdam, The Netherlands, Buster et al^14^ reported a difference in FEV_1_ from predicted values, finding that heroin smokers had an FEV_1_ of 260 mL less than predicted FEV_1_.

The rapid decline in FEV_1_ and the increase in respiratory symptoms in this population suggest heroin smoking is a driver of decline in lung function. Similarly, once established, this decline appears to continue even in people who stop smoking drugs.

Although COPD hospital admissions vary greatly across the United Kingdom, patients with COPD tend to have high health-care usage, particularly in areas of high deprivation.^29^^,^^30^ Previous research has also shown that heroin users with respiratory exacerbations are more likely to be readmitted with exacerbations than current/ex-tobacco smokers (OR, 1.00 vs 0.22/0.26, respectively).^18^ It is also clear that with the high levels of health-care access observed in this population, it is likely that ongoing trends toward inhaling heroin will further increase the use of, and burden on, the health system.^4^^,^^6^

The strengths of our study include that we followed-up the participants over a 18- to 24-month period in a community clinic setting. We have shown that it is feasible to engage this client group in both baseline and follow-up spirometry allowing for a diagnosis to be made. The lost to follow-up rate is a major limitation of this study, reducing the power of statistical analysis and making stratification of our results by age or GOLD stage unfeasible. Given a larger group, this information would potentially be helpful for targeting care, and is an area for future investigation. This population tends to smoke a mix of heroin, crack, and tobacco, establishing a causal relationship with therefore difficult. The participants in the study were generally from a poor socioeconomic background, and there is potential that their living condition environment could contribute to the rate of decline. Without significant heterogeneity of such potentially confounding factors, we have been unable to address this question further. There is also potential for selection bias, with people who regularly attend methadone clinics and have concerns about their respiratory system more likely to participate in the study.

In summary, our findings show the significant respiratory impairment with which heroin smoking is implicated, and a concerning accelerated rate of decline over time. Future studies with larger cohorts, possibly in the context of a targeted public health intervention, are needed to understand if specific subgroups are especially vulnerable, and how the personal and health-care costs associated with chronic respiratory illness could be best averted. The study methodology is in support of it being feasible to colocate respiratory and drug services to one community location. Future studies may benefit from a parallel group of heroin users without spirometric abnormalities at baseline to determine their rate decline compared with patients with COPD. These results combined with previous studies support the call for enhanced screening for inhaled drug users.^19^ A pilot followed by clinical trial would be needed to assess if screening and treatment services would be clinically and cost-effective in this population.

## Supplementary Data

e-Online Data
