# Supplementary material for: Screening Heroin Smokers Attending Community Drug Clinics for Change in Lung Function: A Cohort Study
Source: Chest. 2019 Nov 22;157(3):558–65. doi: 10.1016/j.chest.2019.11.006 (PMC7078587; doi:10.1016/j.chest.2019.11.006)
Supplement: e-Online Data [file mmc1.pdf]

CHEST Online Supplement

Screening Heroin Smokers Attending Community Drug Clinics for Change in Lung Function

A Cohort Study

Rebecca Nightingale, MSc; Kevin Mortimer, MD, PhD; Emanuele Giorgi, PhD; Paul P. Walker, MD; Marie Stolbrink, MD; Tara Byrne, BSc; Kerry Marwood, NVQ; Sally Morrison-Griffiths, MD; Susan Renwick, MPH; Jamie Rylance, MD, PhD; and Hassan Burhan, MD

CHEST 2020; 157(3):558-565

Online supplements are not copyedited prior to posting and the author(s) take full responsibility for the accuracy of all data.

© 2019 AMERICAN COLLEGE OF CHEST PHYSICIANS. Reproduction of this article is prohibited without written permission from the American College of Chest Physicians. See online for more details. **DOI:** 10.1016/j.chest.2019.11.006

e-Table 1: *Characteristics of participants completing follow up questionnaires, with and without ATS standard spirometry*

Variable	Acceptable spirometry N (%) Mean (SD)	Unacceptable spirometry or declined/excluded for medical reasons N (%) Mean (SD)	Chi$_2$ or Ttest p-value
**Sex**	**N=106**	**N=55**	
Female	**30.2 (32)**	**25.5 (14)**	**0.53**
Male	**69.8 (74)**	**74.5 (41)**	
**Age in years, mean (SD)**	**50.9 (5.2)**	**51.3 (5.6)**	**0.67**
**IMD Score**	**50.3 (12.9)**	**53.9 (12.3)**	**0.09**
**Occupation**			**0.65**
Unemployed	**85.9 (91)**	**83.6 (46)**	
Employed	**14.1 (15)**	**16.2 (9)**	
**Housing**			**0.76**
Own home (included rental),n	**77.4 (82)**	**76.4 (42)**	
Homeless, n (%)	**4.72 (5)**	**1.82 (1)**	
Other, n (%)	**17.9 (19)**	**21.78 (12)**	
**Cigarette Smoking Status**			**0.125**
Current , n (%)	**78.3 (83)**	**50 (90.9)**	
Ex, n (%)	**22 (20.8)**	**5 (9.1)**	
Never, n (%)	**1 (0.9)**	**0 (0)**	
**Heroin Smoking Status**			**0.312**
Current, n (%)	**44.34 (47)**	**52.7 (29)**	
Ex, n(%)	**55.7 (59)**	**47.3 (26)**	
**Crack smoking**			**0.74**
Current, n (%)	**18.9 (20)**	**23.6 (13)**	
Ex, n(%)	**55.6 (59)**	**54.6 (30)**	
Never, n (%)	**25.5 (27)**	**21.8 (12)**	

Online supplements are not copyedited prior to posting and the author(s) take full responsibility for the accuracy of all data.
